# Keratin/Copper Complex Electrospun Nanofibers for Antibacterial Treatments: Property Investigation and In Vitro Response

**DOI:** 10.3390/ma17102435

**Published:** 2024-05-18

**Authors:** Maria Laura Tummino, Iriczalli Cruz-Maya, Alessio Varesano, Claudia Vineis, Vincenzo Guarino

**Affiliations:** 1 Institute of Intelligent Industrial Technologies and Systems for Advanced Manufacturing (STIIMA), National Research Council of Italy (CNR), Corso Giuseppe Pella 16, 13900 Biella, Italy; 2 Institute for Polymers, Composites and Biomaterials (IPCB), National Research Council of Italy (CNR), Mostra d’Oltremare, Pad. 20, V. le J.F. Kennedy 54, 80125 Napoli, Italyvincenzo.guarino@cnr.it (V.G.)

**Keywords:** wool keratin, copper, electrospun nanofibers, antibacterial membranes, cytocompatibility, cell growth

## Abstract

The frontiers of antibacterial materials in the biomedical field are constantly evolving since infectious diseases are a continuous threat to human health. In this work, waste-wool-derived keratin electrospun nanofibers were blended with copper by an optimized impregnation procedure to fabricate antibacterial membranes with intrinsic biological activity, excellent degradability and good cytocompatibility. The keratin/copper complex electrospun nanofibers were multi-analytically characterized and the main differences in their physical–chemical features were related to the crosslinking effect caused by Cu^2+^. Indeed, copper ions modified the thermal profiles, improving the thermal stability (evaluated by differential scanning calorimetry and thermogravimetry), and changed the infrared vibrational features (determined by infrared spectroscopy) and the chemical composition (studied by an X-ray energy-dispersive spectroscopy probe and optical emission spectrometry). The copper impregnation process also affected the morphology, leading to partial nanofiber swelling, as evidenced by scanning electron microscopy analyses. Then, the membranes were successfully tested as antibacterial materials against gram-negative bacteria, *Escherichia coli*. Regarding cytocompatibility, in vitro assays performed with L929 cells showed good levels of cell adhesion and proliferation (XTT assay), and no significant cytotoxic effect, in comparison to bare keratin nanofibers. Given these results, the material described in this work can be suitable for use as antibiotic-free fibers for skin wound dressing or membranes for guided tissue regeneration.

## 1. Introduction

The excessive use of antibiotics over the years has led to an increase in antimicrobial resistance, which means that bacteria are able to survive existing synthetic antibiotics, potentially causing sanitary crises [1,2,3]. According to the World Health Organization [4], antimicrobial resistance kills at least 1.27 million people worldwide and was associated with nearly 5 million deaths in 2019. The mechanisms of antimicrobial resistance can be categorized as follows: (i) limiting the uptake of a drug; (ii) modifying a drug’s target; (iii) inactivating a drug; (iv) causing active drug efflux [5]. The availability of novel pharmaceutical substances is essential to resolve this issue; however, antibiotic discovery is particularly difficult given the intrinsic characteristics of bacteria barrier mechanisms: traditional methods of screening environmental isolates or compound libraries have not produced a new drug in over 30 years [6].

Therefore, research in this field has aimed to develop new strategies for the production of biomaterials with antimicrobial activity [7,8]. These new strategies include physicochemical methods for the design of antibiotic-free materials that can be applied in the biomedical field (i.e., tissue engineering, wound dressing, medical devices) [9,10]. Indeed, the properties and application of materials can be changed significantly by the modification of the surface functional groups, the formation of composites with inorganic materials, or the incorporation of other active agents (i.e., metallic ions, novel antibacterial agents, micro- or nano-structures) [11,12,13,14].

From this perspective, metal ions such as Ag^+^, Cu^2+^, and Zn^2+^ can be exploited to disrupt the bacterial cell membrane and intracellular damage caused through the generation of toxic compounds [15]. Regarding copper, on which this work is focused, it has been reported that its positively charged ions can act through (i) damaging pathogens via physical interactions by puncturing the negatively charged membranes of microorganisms, (ii) generating reactive oxygen species (ROS) through a Fenton-like mechanism, or (iii) combining both actions, inducing an oxidative stress response and the involvement of endogenous ROS [16].

Due to its high antimicrobial properties, copper has been immobilized into several materials or used as a surface coating to decrease the formation of biofilms [15,17,18,19,20,21]. Moreover, copper is an element involved in numerous physiological and metabolic processes critical for the appropriate functioning of almost all tissues in the human body [22,23,24]. Therefore, it is suitable for integration with the biomaterials used for tissue engineering to combine the main properties of both material types. For instance, Cu^2+^ ions have been mixed into a bioactive glass or incorporated into hydrogels for use in tissue engineering and wound dressing [25,26,27].

In recent years, the use of biomass products has led to the development of materials, particularly polysaccharide-based (i.e., sodium alginate, cellulose, chitin, starch) and protein-based (i.e., silk, sericin, zein, keratin, soy protein) materials, with good biocompatibility [28,29,30,31,32,33,34,35]. Among them, wool keratin is a natural animal fiber protein that can be extracted from by-products of the textile industry and used to fabricate materials for biomedical applications [36,37,38,39]. Besides its good biocompatibility, keratin contains the amino acid sequences of leucine–aspartic acid–valine (LDV), arginine–glycine–aspartic acid (RGD), and glutamic acid–serine (EDS), which are recognized by their cell surface integrin receptors (i.e., α_4_β_1_ integrin), which are able to promote cell adhesion with the extracellular matrix (ECM) in native tissues [40,41,42]. Moreover, it is possible to regulate tissue homeostasis and skin wound healing by recognizing these amino acid sequences through the β_1_ integrin in the membrane of fibroblasts [43,44]. Therefore, different keratin-based materials have been designed (i.e., films, hydrogels, sponges, and fibers) for various applications in tissue engineering [38,45,46,47,48]. In particular, electrospun fibers of wool keratin have been fabricated to mimic the extracellular matrix of tissues by providing morphological (i.e., fiber morphology and alignment), and biochemical cues (i.e., binding motifs) to support cell growth [49,50,51]. Keratin fibers have also shown a slight antimicrobial activity that could be influenced by two factors: the physical factor, related to the direct contact of bacterial cells, which can lead to the disruption of the bacterial cell membrane [52,53], or the secondary structure of keratins (i.e., alpha-helix, disulfide bonds, amino acid, and carboxylic groups) with no outstanding antimicrobial activity compared with other materials [54,55]. However, the antimicrobial properties of keratin-based materials (i.e., modified wool or keratin nanofibers) can be noticeably improved through the addition of metal ions, such as Ag^+^ and Cu^2+^ ions [56,57]. Another aspect to be considered, though, is that the use of metal ions can have some operative limitations when a high level of release occurs [56].

Hence, in this work, waste-wool-derived keratin nanofibers were produced by electrospinning and blended with copper using a simple impregnation procedure after a preliminary optimization step. The keratin/copper complex electrospun nanofibers were then characterized to validate their use in the biomedical field. To the best of the authors’ knowledge, this is the first time that similar materials have been prepared and subjected to this kind of study.

## 2. Materials and Methods

**Figure 1:**
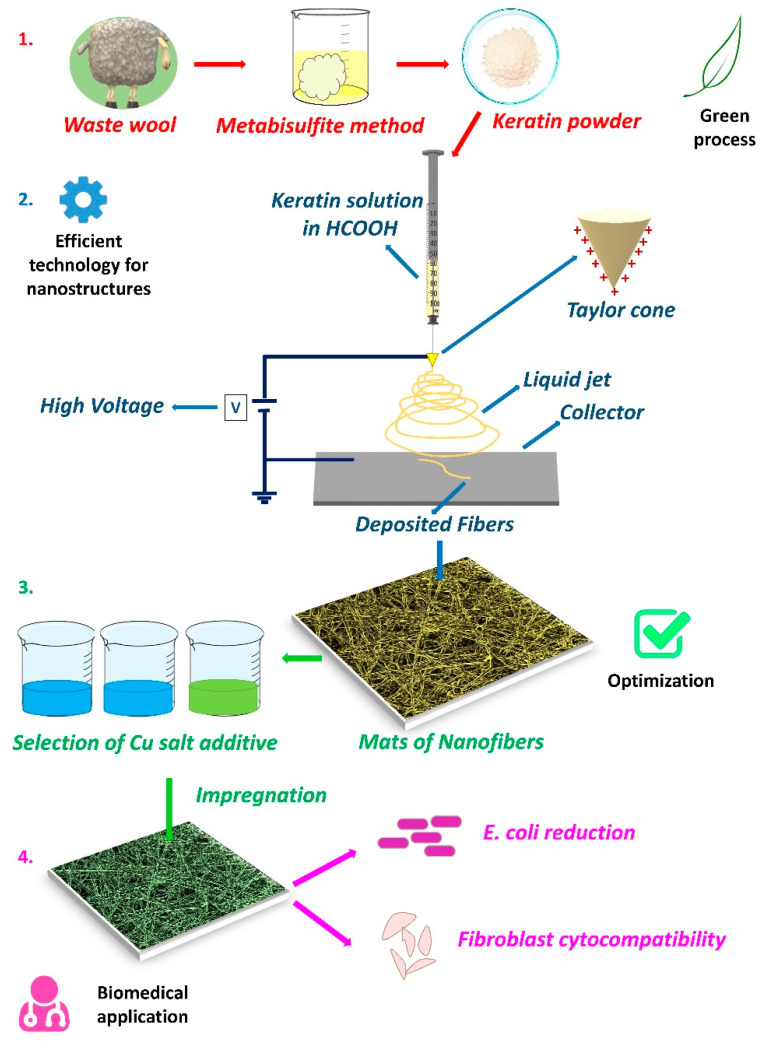
Representation of the relevant steps forming the basis of this work: the sustainable extraction of keratin from waste wool (**1**); the electrospinning technique model used to produce nanometric fibers enclosing the main components constituting the equipment/process (**2**); the choice of proper copper salt and its subsequent characterization for the material optimization (**3**); tests regarding its use as antibacterial agent and verification of its cytocompatibility, in a preliminary assessment, for real applications (**4**).

A schematic representation of the workflow forming the basis of this study is depicted in Figure 1, where the green aspects, the technological facets, and the significance of the application are displayed. A simplified model of the electrospinning apparatus and its mechanism has been reported.

### 2.1. Preparation of Keratin/Copper Complex Electrospun Nanofibers

Waste wool fibers were used to obtain keratin powder through sulfitolysis, as previously reported [58]. Briefly, a wool sample was cleaned by Soxhlet with petroleum ether to eliminate fatty matter, washed with distilled water, and conditioned at 20 °C, 65% relative humidity (RH), for 24 h. After that, the clean and conditioned wool fibers were cut into snippets and treated with 100 mL of a solution containing urea (8 M) and sodium metabisulfite (0.5 M); then, they were adjusted to pH 6.5 with sodium hydroxide (5 M) and subjected to shaking for two hours at 65 °C. The mixture was filtered through a 5 µm pore-size filter and dialyzed against distilled water in a cellulose tube (molecular cut-off 12–14 kDa) for four days, repeatedly changing the distilled water. Finally, the resulting purified liquid was freeze-dried [59].

Solutions of keratin powder in formic acid (15 wt.%) were employed to produce nanofiber (NFs) membranes using a single-jet electrospinning prototype (see Figure 1) [52,60]. This consisted of a flat plate collector (in this case, covered by polypropylene nonwovens TNT 17 gsm), a KDS 200 high-precision syringe pump (KD Scientific Inc., Hill Road Holliston, MA, USA), a stainless-steel needle (d = 0.4 mm), and an SL50 high-voltage generator (Spellman, Broomers Hill Ln, Pulborough, UK). The working conditions to produce NFs-membranes were as follows: voltage 25 kV, tip-collector distance 15 cm, flow rate 0.003 mL min^−1^, temperature 23.0 ± 0.7 °C, and humidity 25.2 ± 1.7% RH.

A reference sample was post-treated to prevent the water solubilization of keratin proteins by thermal treatment, particularly through heating the keratin nanofibrous membrane in an oven for 2 h at 180 °C in air and thus inducing crosslinking (Keratin_TT).

The other samples were, instead, subjected to chemical modifications carried out through nanofibrous membrane impregnation in different copper salt solutions. The solutions were prepared in ethanol (≥99.8%, Sigma Aldrich, Milan, Italy), as this solvent is able to dissolve the salts but not the keratin NFs. The solutions were as follows:

(a) 5 wt/v% copper(II) nitrate trihydrate, Cu(NO_3_)_2_·3H_2_O (CAS 10031-43-3, Sigma-Aldrich, Merck Life Science S.r.l., Milano, Italy);
(b) 1 wt/v% copper (II) acetate Cu(CH_3_COO)_2_ (CAS 142-71-2, Sigma-Aldrich, Merck Life Science S.r.l., Milano, Italy);
(c) 5 wt/v% copper(II) chloride, CuCl_2_ (CAS 7447-39-4, Sigma-Aldrich, Merck Life Science S.r.l., Milano, Italy).

The nanofibers were soaked for 24 h; then, the copper solution was withdrawn, and the materials were rinsed with fresh ethanol. Moreover, in order to carefully remove the excess salt, the membranes were immersed for 6 days in pure ethanol (changing the ethanol three times).

### 2.2. Material Characterization

The morphology of membranes was examined using an EVO10 Scanning Electron Microscope (SEM, Carl Zeiss Microscopy GmbH, Oberkochen, Germany) with an acceleration voltage of 20 kV. The samples were sputter-coated with a 20 nm thick gold layer in rarefied argon (20 Pa), using a SC7620 Sputter Coater (Quorum, East Sussex, UK)

For the elemental analysis, X-ray energy-dispersive spectroscopy (EDX, Genesis 2000i, Mahwah, NJ) analysis was used to evaluate the composition, and specifically to detect the presence of Cu in the fibers.

Attenuated total reflectance Fourier transform infrared (ATR-FTIR) spectra were recorded with a Thermo Nicolet iZ10 spectrometer (Milan, Italy) equipped with a ZnSe crystal in the range 4000–650 cm^−1^, with 32 scans and 4 cm^−1^ band resolution.

The thermal behavior of the samples was investigated by thermogravimetric analysis (TGA) and differential scanning calorimetry (DSC). For TGA analyses, (Mettler Toledo TGA-DSC 1, Schwerzenbach, Switzerland), about 5 mg of the sample within an alumina pan was heated from 30 °C to 800 °C at a rate of 10 °C min^−1^ in N_2_ flow, 70 mL min^−1^. Derivative thermogravimetry (DTG) was used to identify the temperature of maximum mass-loss rates. Differential scanning calorimetry (DSC) was carried out with a DSC calorimeter (Mettler Toledo 821e, Schwerzenbach, Switzerland) calibrated by an indium standard. The calorimeter cell was flushed with 100 mL min^−1^ nitrogen. The run was performed from 30 to 500 °C at the heating rate of 10 °C min^−1^. The data were processed using the STARe Software (version 9.30).

The test for Cu release in water was evaluated by putting the dried nanofibrous membrane of Keratin_Cu in distilled water and letting it stir for 1 h (time chosen in correspondence with the contact time necessary for the antibacterial trials). The resulting aqueous solution was analyzed by an Inductively Coupled Plasma Optical Emission Spectrometer (ICP-OES, Optima 7000 DV, Perkin Elmer, Waltham, MA, USA), and the concentration of the released copper ions was determined by means of a calibration curve [61]. The “water-washed” material was recovered as well and labeled Keratin_Cu_H_2_O.

### 2.3. Antibacterial Tests

The antimicrobial activity was evaluated according to the ASTM E 2149-2013 [62] procedure “Standard test method for determining the antimicrobial activity of immobilized antimicrobial agents under dynamic contact conditions”. This method is a quantitative procedure. The bacteria used were *Escherichia coli* ATCC 11229 (gram-negative).

The bacteria were grown in a proper nutrient broth (buffered peptone water for microbiology, VWR Chemicals, Milan, Italy) for 24 h at 37 °C. The bacteria concentration was measured with a spectrophotometer and diluted into a sterile buffer to reach a 1.5–3.0 × 10^5^ CFU/mL working dilution. This bacterial inoculum was placed in contact with the antibacterial agent (Keratin_Cu and Keratin_Cu_H_2_O membranes) under shaking at room temperature for 1 h. Given the strong antimicrobial activity of copper, the antibacterial agent/inoculum ratio was modified from the standard 1 g/50 mL to a more challenging 1 g/500 mL [2]. After the contact time had passed, 1 mL of inoculum was diluted 1000 times and plated in Petri dishes with Yeast Extract Agar (Sigma Aldrich). The Petri dishes were incubated for 24 h at 37 °C, and then the surviving bacteria colonies were counted and compared to the initial bacteria concentration of the inoculum to calculate the percentage of bacterial reduction using the following equation (Equation (1)):

(1) $$Reduction\left(\%\right)=\frac{\left(A-B\right)\times 100}{A}$$

where A is the number of viable microorganisms before treatment and B is the number of viable microorganisms after treatment.

### 2.4. Cell Culture

For in vitro assays, the L929 cell line (fibroblasts derived from mouse, Sigma-Aldrich, St. Louis, MO, USA) was used. L929 cells were cultured in a 75 cm^2^ cell culture flask with Dulbecco’s Modified Eagle Medium (DMEM, Sigma-Aldrich, Milan, Italy), supplemented with 10% of fetal bovine serum (FBS, Sigma-Aldrich, St. Louis, MO, USA), antibiotic solution (streptomycin 100 μg/mL and penicillin 100 U/mL, Sigma-Aldrich, Milan, Italy), and 2 mM of L-glutamine (Sigma-Aldrich, Milan, Italy) until reaching a confluence of 80%. The environmental conditions were 37 °C in a humidified atmosphere with 5% CO_2_ and 95% air.

### 2.5. Cytocompatibility Assays

Cell adhesion and proliferation were determined using the Cell Proliferation Kit II (XTT, Roche Diagnostics Deutschland GmbH, Mannheim, Germany, purchased by Sigma-Aldrich). For in vitro assays, all the samples were circularly cut (e.g., specimen area of about 0.3 cm^2^) and placed in a 96-well cell culture plate. For cell adhesion, at 4 and 24 h, L929 cells were seeded in a density of 2 × 10^4^ cells/well. After this time, samples were washed two times to remove the unattached cells and a solution of medium with XTT was added to incubate for four hours. After the incubation time, the supernatant was recovered and placed in a 96-well plate reader. Absorbance measurements were recorded at 450 nm with a plate reader (Wallac Victor 1420, PerkinElmer, Boston, MA, USA). Results of cell adhesion are presented as a percentage of adhesion out of the tissue culture plate (TCP).

In order to evaluate the cell–material interaction, Cell-Tracker Green CMFDA (5-chloromethyl–fluorescein diacetate, Invitrogen by Thermo Fisher Scientific, Monza, Italy) was used for observation via confocal microscopy. Briefly, cells were seeded (1.5 × 10^4^ per well) onto the fibers. After 24 h in cell culture, the medium was replaced with serum-free and red phenol-free DMEM and incubated in standard conditions for 1 h. Then, samples were washed with Phosphate Buffered Saline (PBS) and incubated in a complete medium in standard conditions for 24 h. Cells were fixed with 4% of paraformaldehyde (PFA) and stained with 1 μg mL^−1^ 4′,6-diamidino-2-phenylindole (DAPI) for 5 min before imaging. The cell morphology and cell interactions of L929 cells were evaluated by confocal microscopy.

For cell proliferation, L929 cells were seeded onto fibers at a density of 1 × 10^4^ per well and incubated under standard conditions. After 1, 3, 7, and 14 days of incubation, an XTT assay was performed. After the cell culture period, the medium was replaced by fresh media containing an XTT working solution, according to the manufacturer’s instructions, and incubated for 4 h. XTT assay is based on the cleavage of the yellow tetrazolium salt XTT to form a soluble orange formazan dye using living cells. After the incubation time, the supernatant was recovered and placed in a 96-well plate reader to measure the absorbance at 450 nm with a plate reader (Wallac Victor 1420, PerkinElmer, Boston, MA, USA). The experiments were conducted in triplicate. During the experiment, the cell culture media was changed every two days and replaced with fresh media.

The indirect method was used to evaluate the cytotoxic effect of the content and release of molecules from fibers. L929 cells were seeded in a 48-well cell culture plate at a concentration of 2 × 10^4^ cells/well and incubated with standard cell culture media in standard environmental conditions. After the cells reached confluence, the medium was removed and replaced with 200 μL of serum-free medium DMEM recovered from fibers (ca. 1.0 mg mL^−1^) incubated from 10 min to 48 h. After 24 h of incubation with the medium from fibers, the medium was removed to perform an XTT assay, as described above, which indicated the viable cells. Cells incubated with serum-free DMEM served as positive control.

For in vitro assays, the results were presented as mean standard deviation *(n* = 3). An analysis of variance (ANOVA) was performed, followed by Tukey’s post hoc. A value of *p* < 0.05 was considered to determine statistically significant differences.

## 3. Results and Discussion

### 3.1. Preliminary Morphological Screening of Different Treatments’ Influence on Keratin NFs

**Figure 2:**
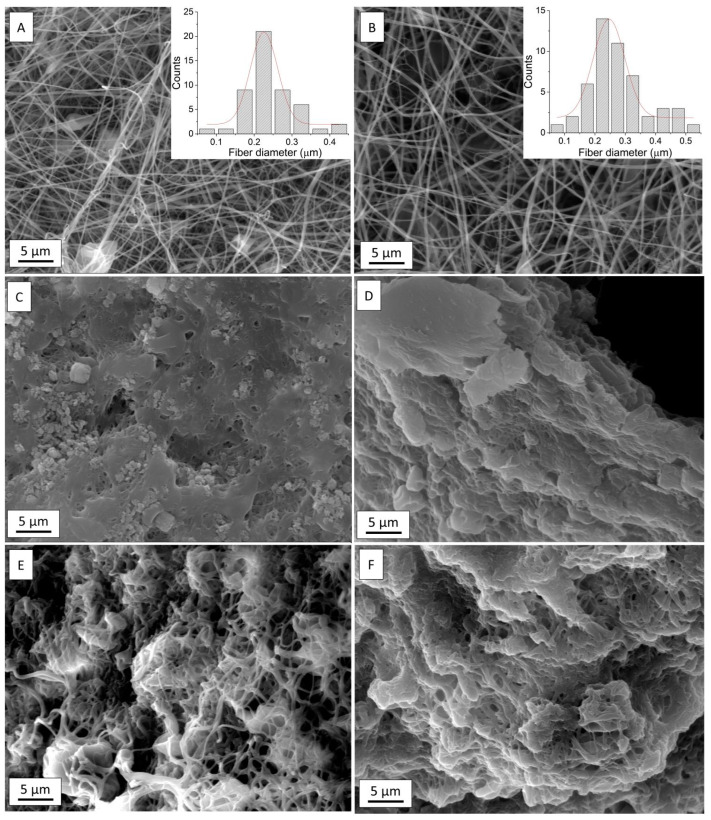
SEM images of various samples: (**A**) as-prepared electrospun keratin NFs; (**B**) electrospun keratin NFs, thermally treated (Keratin_TT); (**C**) electrospun keratin NFs, treated with Cu(NO_3_)_2_·3H_2_O; (**D**) electrospun keratin NFs, treated with Cu(CH_3_COO)_2_; (**E**) electrospun keratin NFs, treated with CuCl_2_ (Keratin_Cu); (**F**) electrospun keratin NFs, treated with CuCl_2_ after 1 h water soaking (Keratin_Cu_H_2_O). Insets in (**A**,**B**) are related to the distribution of NF diameters.

The electrospun keratin NFs treated with Cu(NO_3_)_2_·3H_2_O, Cu(CH_3_COO)_2_ and CuCl_2_ were analyzed by SEM, whose images are reported in Figure 2 (respectively, C, D, E), compared with the as-prepared keratin NFs (Figure 2A) and the thermally treated ones (Keratin_TT, Figure 2B). As-prepared keratin NFs and Keratin_TT showed the typical shape of electrospun nanofibers with a mean diameter of 243 ± 66 nm and 276 ± 95 nm, respectively [59,60,63]. All the Cu-added samples were resistant to both alcoholic and water solutions, proving that copper created chemical links and insoluble, stable systems. However, among the Cu-containing membranes, the less compromised nanofibrous network is the one derived from the copper chloride treatment, which caused only partial NF swelling (with an average fiber diameter increase of ca. 30%) and the formation of agglomerates [64,65,66]. Thereby, subsequent studies have focused on this type of membrane, which is indicated as Keratin_Cu. For the sake of completeness, to mimic a more realistic environment, Keratin_Cu_H_2_O was also investigated. This sample showed a more pronounced swelling of the fibers, possibly due to the inherent increase in the interaction of sulfitolysis-prepared keratin with water compared to ethanol [67,68,69] (Figure 2F).

### 3.2. Compositional and Physical–Chemical Characterization

**Figure 3:**
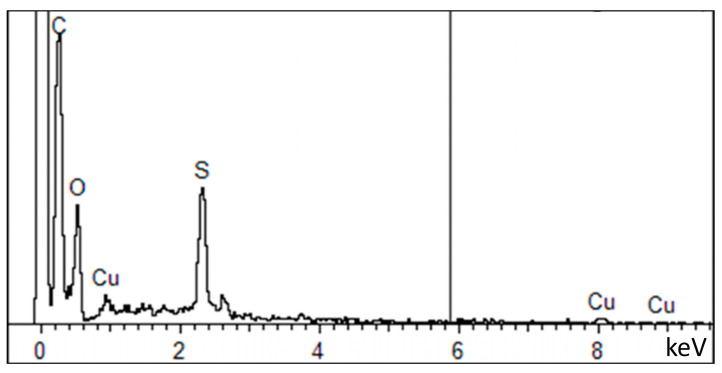
EDX elemental analysis of Keratin_Cu fibers.

The EDX results showed the presence of sulfur, ascribed to the significant amount of cysteine in keratin, and confirmed the presence of copper in the fibers (Figure 3). In detail, the ratios between the weight percentage of S and Cu out of that of carbon were calculated as follows: S/C = 0.092 (in good accordance with the literature [70]) and Cu/C= 0.013.

**Figure 4:**
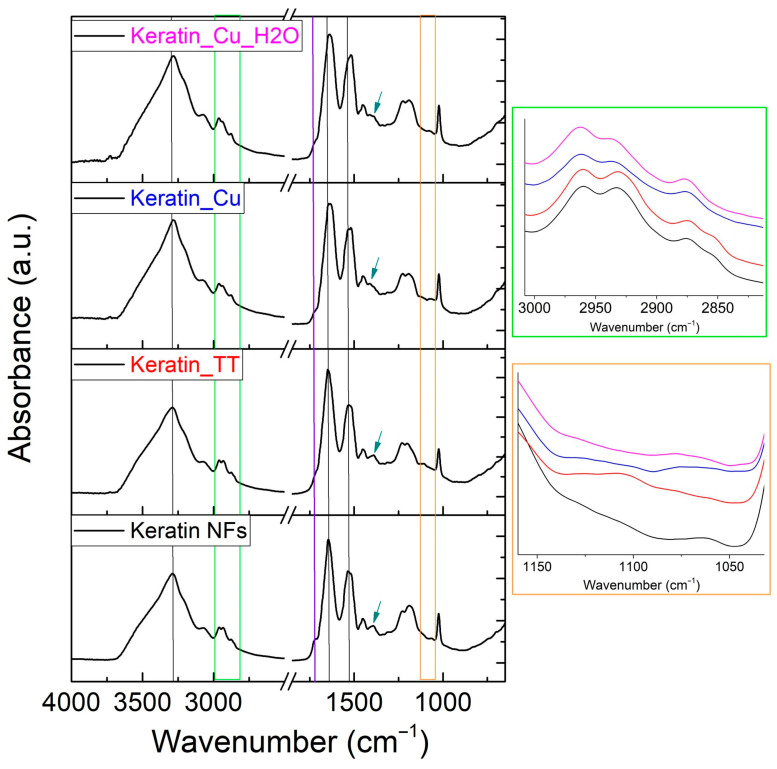
ATR-FTIR results for the different samples considered in this work. On the left, the whole spectra are presented (black and violet lines and dark cyan arrows indicate particular regions described in the main text); on the right, two magnified spectral areas are presented in the ranges 3000–2800 cm^−1^ (green framework) and 1160–1040 cm^−1^ (orange framework).

The vibrational features of the materials were investigated through ATR-FTIR spectroscopy (Figure 4). The typical signals of keratin were detected for all the samples. The main absorption bands are related to the N–H stretching mode (Amide A) centered at ca. 3300 cm^−1^, C=O stretching (Amide I) at 1640 cm^−1^, C–N stretching, and N–H in-plane bending vibrations (Amide II) at 1530 cm^−1^ [50,71]. At 1453 cm^−1^, additional vibrations of amino groups and alkyl side chains in proteins are visible [72,73,74,75]. In the 2950–2860 cm^−1^ region, CH_3_ and CH_2_ asymmetric and symmetric vibrations can be found [76]. The Amide III peak occurs at around 1200 cm^−1^ with a complex signal that indicates an in-phase combination of C-N stretching and N-H in-plane bending, with some contribution from C-C stretching and C=O bending vibrations [50,71,77,78]. The peak at 1025 cm^−1^ is related to the S-O symmetric stretching vibrations of cysteine-S-sulfonate residues (Bunte salts) derived from the extraction of protein from wool [78].

Furthering the differences among the samples, the first phenomenon to underline is that the shoulder at 1720 cm^−1^ (violet line, Figure 4) decreased because of the heating treatment and copper modification. This shoulder was attributed to the stretching vibrations of the C=O bonds of the terminal-free carboxylic groups of the protein and the side-chain carboxylic groups in amino acids, such as glutamic acid and aspartic acid. As previously reported, the high-temperature treatment could induce a crosslinking reaction involving acid (e.g., glutamic and aspartic) and base (e.g., arginine) side-chain groups of amino acids [63]. Similarly, Cu^2+^ could be complexed by carboxylic groups, acting as a crosslinker. Such new links could also be the cause of the smoothing and the shift to lower wavenumbers for the Amide I and Amide II peaks for Keratin_TT and, more significantly, for Cu-containing membranes. This phenomenon is observable in Figure 4 (where black lines correspond with those peaks and a reference line denotes the Amide A band) and is correlated with a change in the strength of the molecular interactions and protein conformations [50,79].

Another area that is particularly sensitive to changes in the protein’s secondary structure, the nature of the side-chain groups, and the hydrogen bonding is that of Amide III (around 1200 cm^−1^). Indeed, in the present study, the as-prepared keratin NFs are differentiated from the other samples due to the different proportions between the “humps” at 1185 and 1230 cm^−1^, again indicating the diverse behavior between crosslinked and non-crosslinked keratin-based membranes [63]. The dark cyan arrows of Figure 4 point to the net intensity reduction in the signal at about 1390 cm^−1^ with respect to the adjacent peak in the case of Cu-containing samples. This IR mode was previously associated with the symmetric in-plane bending vibrations of two adjacent CH_3_ groups in the amino acid side chains [50] and its decrement could be another index of the protein’s conformational modification, which limited the CH_3_ exposition/vibration. This hypothesis seems to be confirmed by the analysis of the 3000–2800 cm^−1^ region (green framework, Figure 4). If that spectral portion for as-prepared keratin NFs and Keratin_TT was superimposa-ble, Keratin_Cu and Keratin_Cu_H_2_O showed the disappearance of the shoulder at 2853 cm^−1^ near the peak at 2873 cm^−1^, related to symmetric stretching of methylene [80,81].

Lastly, the range 1160–1040 cm^−1^ (orange framework, Figure 4) is presented since, according to Jiang et al. [71], it is representative of the S-O fingerprint but can also be attributed to C-O stretching [82] and, therefore, could be indicative of the oxidation state, the degree of disulfide bond cleavage in the samples, and the interaction between SO_3_^−^ and Cu^2+^ [83,84].

**Figure 5:**
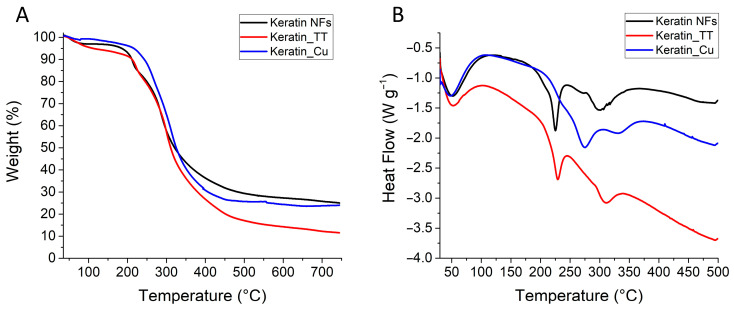
Thermal analyses: (**A**) TGA and (**B**) DSC.

In Figure 5 and Table 1, the thermal analysis results are reported. Qualitatively, the TGAs (Figure 5A) of all the samples are characterized by the first phenomenon of water evaporation up to 200 °C. The steepest trend was found for as-prepared Keratin_TT. Secondly, Keratin NFs and Keratin_TT demonstrated a visible thermal-induced event between 210 and 230 °C, which corresponded to the values of the first peak of the DTG curves (Table 1). The same event was “hidden” in the TGA curve for Keratin_Cu, but it was detected at higher temperatures (>40 °C) by the DTG analysis. The major weight loss for keratin-based samples was centered around 300 °C, but again, the temperature rose in the order Keratin NFs < Keratin_TT < Keratin_Cu, confirming the higher thermal stability of the Cu-added sample. Similar trends were shown by DSC analyses (Figure 5B and Table 1). The thermal phenomena that occurred can be attributed to the denaturation of keratin secondary structures and the degradation of the polypeptide backbone with the release of volatile compounds, such as CO, H_2_S, CH_4_, and HCN [85,86].

Regarding the TGA of the final residues, it is clear that Keratin_TT underwent a higher weight loss than Keratin NFs and Keratin_Cu, whose results were comparable. The reason for Keratin_TT’s behavior is likely (i) the degradation of heat-sensitive amino acids and the production of water through the reaction of amine and carboxyl groups during thermal treatment; (ii) the difference in the ratio between the heat-resistant and heat-labile amino acids in thermally treated keratin nanofibers; and (iii) the formation of volatile molecules as products of condensation reactions [63].

### 3.3. Antibacterial Tests

**Figure 6:**
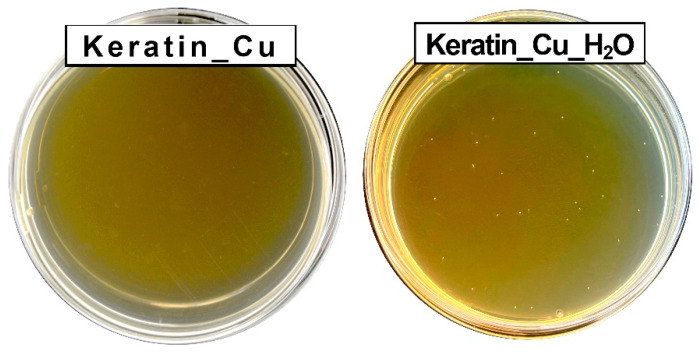
Growth of *E. coli* colonies in Petri dish after treatment with Keratin_Cu and Keratin_Cu_H_2_O.

*Escherichia coli* has been used as a model bacteria due to its sensitivity to copper ions related to bacterial cell wall peptides [87] and cytoplasmic damage [88]. As is visible in Figure 6, the antibacterial performance of Keratin_Cu against *E. coli* reached complete bacterial removal (100%), and this outcome was brought about by the presence of copper, since keratin fibers alone did not demonstrate any significant activity. The sample Keratin_Cu_H_2_O, instead, resulted in a 67% bacterial reduction. This fact was hypothesized to be correlated with the slight loss of Cu ions in the water solution [89], where the eventual residues of excess copper salt were solubilized, although repeated washings in ethanol. Indeed, the solubility of CuCl_2_ in ethanol is reported as 530 g/L at 15 °C while, in water, 620 g/L at 20 °C was reported. In order to determine the extent of this phenomenon, ICP-OES was performed on the aqueous solution after 1 h contact with Keratin_Cu, showing that, for each mg of the membrane, ca. 3.5 μg was released.

### 3.4. In Vitro Assays

**Figure 7:**
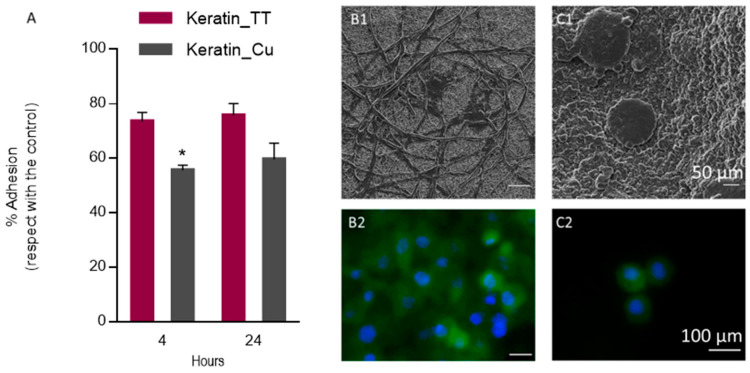
(**A**) Cell adhesion results at 4 and 24 h after cell culture. Results represent the percentage of cell adhesion with respect to the control (TCP) (* *p* < 0.05). SEM (scale bar: 50 μm), and confocal (scale bar: 100 μm, DAPI: blue; cell-tracker: green) images of L929 cells seeded onto Keratin_TT (**B1**, **B2**), and Keratin_Cu fibers (**C1**,**C2**) after 24 h.

In vitro studies were performed to validate the biological response of keratin/copper complex electrospun nanofibers. In recent years, several studies have investigated the cytocompatibility of keratin-based biomaterials and their ability to support cell adhesion and proliferation [50,90]. It was proved that the addition of Cu plays a relevant role in bone metabolism [91] and increases the wound healing rate [27]. Nevertheless, it is well known that an excess of Cu may induce damage in several organs and cellular toxicity through the oxidative pathways [92,93]. Hence, it is important to evaluate the cytotoxic effect of Cu addition in keratin fibers. From the cell adhesion results (Figure 7A), it is possible to observe cells’ stronger preference to adhere to Keratin_TT fibers (around 80% of cell adhesion) due to the presence of cell-binding motifs that are able to improve the cell adhesion [41,54]. In the first 4 h, nearly 60% of cells attached to Keratin_Cu fibers. Moreover, SEM microscopy showed that the structure of Keratin_TT fibers was preserved after 24 h (Figure 7B1), differently from Keratin_Cu fibers, which presented agglomerates and swollen NFs, thus influencing the morphology of cells (Figure 7C1). From the confocal images, it is possible to notice that cells spread along the Keratin_TT fibers with a higher density (Figure 7B2) compared to Keratin_Cu fibers, where the cells, although attached, preferentially remained with a rounded morphology (Figure 7C2).

**Figure 8:**
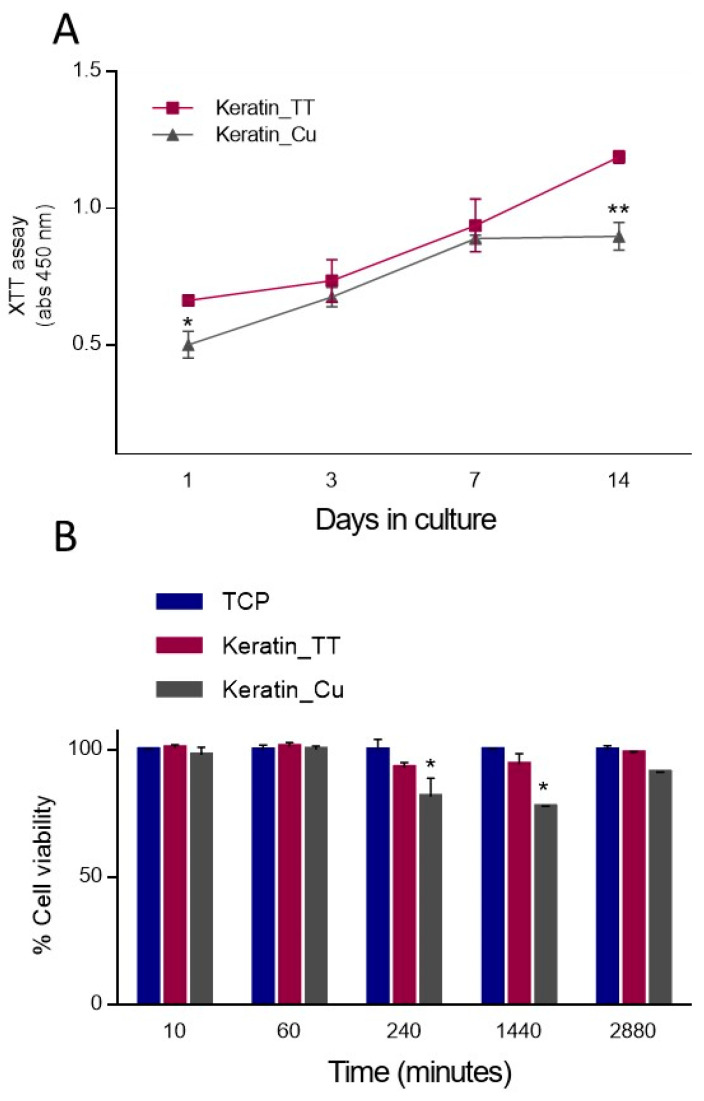
(**A**) Cell proliferation of L929 cells into Keratin_TT and Keratin_Cu fibers. Statistically significant differences are indicated as * *p* < 0.05; ** *p* < 0.01; (**B**) indirect cytotoxicity (significant differences are indicated as * *p* < 0.05).

In particular, Keratin_Cu fibers showed the lowest cell viability at 1 and 14 days with respect to Keratin_TT fibers (Figure 8A). However, a constant increase in cell proliferation was detected for both groups throughout the cell culture period, elucidating the cytocompatibility of the Cu content, which contributed to obtaining a material with synergic properties, as reported elsewhere [94].

The cytotoxicity is usually attributable to the high concentration of Cu^2+^ ions and their release kinetics [95,96,97]. The results of indirect cytotoxicity, performed by incubating the cells with the supernatant recovered from fibers incubated at different times, are reported in Figure 8B. XTT assay demonstrated that the release of substances from the fibers did not affect the cell viability from 10 min to 1 h. However, the release of chemicals from Keratin_Cu at 4 h (240 min) and 24 h (1440 min) showed a cytotoxicity effect when the fiber medium was in contact with cells. After 48 h (2880 min), the cells incubated with Keratin_Cu medium showed a non-significant cytotoxic effect compared to Keratin_TT fibers. The slight cytotoxic effect on L929 cells in the first 24 h can be ascribed to the initial burst release from Cu-loaded keratin fibers, which may induce the production of reactive oxygen species [98,99].

Looking at the literature to contextualize our results in a broader scenario, most of the uses of antibacterial materials functionalized with copper imply the synthesis of Cu or Cu oxides’ nanoparticles [100,101], and only some of these works reported biocompatibility assays [102,103]. Moreover, a direct comparison with the performances of other materials could be deceitful, considering that different antibacterial methods and related quantifications are adopted [104], i.e., the disk diffusion method [105]. Prabhakar et al. [106] developed different antibacterial nanocoatings for textiles based on impregnated copper. The cotton fabrics, after functionalization with Cu(NO_3_)_2_ and Cu nanoparticles with or without synergy with graphene oxide, showed excellent antimicrobial properties against both *Staphylococcus aureus* and *Pseudomonas aeruginosa* bacterial strains. The CuCl_2_-impregnated sample also showed good inhibition zone values. If these promising results are consistent with the outstanding properties of copper-based antimicrobial materials, as also evidenced in our work, in [106], the stability of the material after washing was achieved through the contribution of the polydopamine, used as a glue (a mussel-inspired strategy) that bound the textile substrate and the functionalizing copper. Certainly, the presence of polydopamine implies the addition of reactants and synthetic steps, but it could inspire for future studies looking to enhance the stability of keratin/copper complex materials.

## 4. Conclusions

Keratin/copper complex electrospun nanofibers were prepared for use as antibacterial materials in biomedical applications. The obtained membranes were characterized (showing the physical–chemical influence of copper binding onto the keratin nanofiber network) and were successfully employed in the reduction in *E. coli* bacteria. The effect of copper was also evaluated in terms of biocompatibility (i.e., cell adhesion and proliferation) and cytotoxic effect: the good overall cytocompatibility levels that were assessed are encouraging, although perfectible, outcomes, ensuring the correct balance between antibacterial activity and toxicity would be obtained in the presence of antimicrobial agents like copper. Future studies will be focused on widening the application of such materials against other bacterial strains. Electrospinning could offer the possibility of designing antibiotic-free fibers that can be used as a skin wound dressing or membranes for guided tissue regeneration in periodontal treatments, due to the synergic effect of keratin and copper, which have bioactive and antibacterial properties.

## Figures and Tables

**Table 1:** Main results derived from TGA and DSC. The data are affected by a standard deviation ≤10%.

Parameter	Keratin NFs	Keratin_TT	Keratin_Cu
TGA residue (wt%)	25	12	24
DTG peak 1 (°C)	213	222	266
DTG peak 2 (°C)	286	300	314
DSC peak 1 (°C)	225	229	276
DSC peak 2 (°C)	300	310	331

## Data Availability

Data are contained within the article.

## Abbreviations

ANOVA	Analysis of variance
ATR-FTIR	Attenuated total reflectance Fourier transform infrared spectroscopy
CMFDA	5-chloromethyl-fluorescein diacetate
DAPI	4′,6-diamidino-2-phenylindole
DMEM	Dulbecco’s modified eagle medium
DSC	Differential scanning calorimetry
DTG	Derivative thermogravimetry
ECM	Extracellular matrix
EDS	Glutamic acid–serine
EDX	X-ray energy dispersive spectroscopy
FBS	Fetal bovine serum
ICP-OES	Inductively coupled plasma optical emission spectrometry
LDV	Leucine–aspartic acid–valine
NFs	Nanofibers
PBS	Phosphate-buffered saline
PFA	Paraformaldehyde
RGD	Arginine–glycine–aspartic acid
RH	Relative humidity
ROS	Reactive oxygen species
SEM	Scanning electron microscopy
TCP	Tissue culture plate
TGA	Thermogravimetric analysis
TT	Thermal treatment
